# Supplementary material for: Effects of structurally distinct human HDAC6 and HDAC6/HDAC8 inhibitors against S. mansoni larval and adult worm stages
Source: PLoS Negl Trop Dis. 2024 Feb 28;18(2):e0011992. doi: 10.1371/journal.pntd.0011992 (PMC10927086; doi:10.1371/journal.pntd.0011992)
Supplement: S1 Table — aSASA predicted the total solvent accessible surface (range or recommended value for 95% of known drugs 300–1000); bQPlogP predicted octanol/water partition coefficient (range or recommended value for 95% of known drugs -2–6.5); cQPlogS predicted aqueous solubility in mol/dm3(range or recommended value for 95% of known drugs -6.5–0.5); dQPPCaco predicted apparent Caco-2 cell permeability in nm/sec (range or recommended value for 95% of known drugs >500 great); eQPPMDCK predicted apparent MDCK cell permeability in nm/sec (range or recommended value for 95% of known drugs >500 great); fQPlogHERG predicted IC50 values for blockage of HERG K+ channels (range or recommended value for 95% of known drugs below -5); g%HOA predicted human oral absorption on 0 to 100% scale (range or recommended value for 95% of known drugs >80% high). Range or recommended values are reported in QikProp user manual. *Cmpd name reported in Relitti et al [25]. (DOCX) [file pntd.0011992.s002.docx]

**S1 Table.** Predicted drug-like features for NF2836, NF2838 and NF2839 compounds.

| **Cmpd** | **SASA^a^** | **QPlogP^b^** | **QPlogS^c^** | **QPPCaco^d^** | **QPPMDCK^e^** | **QPlogHERG^f^** | **%HOA^g^** |
| --- | --- | --- | --- | --- | --- | --- | --- |
| **NF2836 (8*)** | 684.14 | 3.01 | -5.04 | 264.54 | 117.53 | -5.45 | 87.94 |
| **NF2838 (7k*)** | 849.77 | 3.29 | -4.58 | 56.07 | 24.31 | -7.83 | 64.57 |
| **NF2839 (7g*)** | 858.76 | 4.16 | -5.27 | 85.16 | 38.19 | -8.03 | 72.90 |
