# Supplementary material for: Effects of structurally distinct human HDAC6 and HDAC6/HDAC8 inhibitors against S. mansoni larval and adult worm stages
Source: PLoS Negl Trop Dis. 2024 Feb 28;18(2):e0011992. doi: 10.1371/journal.pntd.0011992 (PMC10927086; doi:10.1371/journal.pntd.0011992)
Supplement: S2 Table — The IC50(μM) shown was calculated by dose-response curves of the compounds on murine L929 and human BJ fibroblast cells. The range of concentrations were for NF2836, NF2838, NF2839: 100–0.78 μM; and for GA 10–0.078 μM. The data are representative of 2 independent experiments. Gambogic acid (GA) and vehicle (DMSO) were used as positive and negative controls respectively. (DOCX) [file pntd.0011992.s003.docx]

**S2 Table.** Cytotoxicity for NF2836, NF2838 and NF2839 compounds on BJ and L929 fibroblast cell lines.

| **Cells** | **NF2836** | **NF2838** | **NF2839** | **GA** |
| --- | --- | --- | --- | --- |
| **L929** | >100 | 64-100 | 72-73 | 0.5 |
| **BJ** | >100 | >100 | >100 | 0.35 |
