# Supplementary material for: Effects of structurally distinct human HDAC6 and HDAC6/HDAC8 inhibitors against S. mansoni larval and adult worm stages
Source: PLoS Negl Trop Dis. 2024 Feb 28;18(2):e0011992. doi: 10.1371/journal.pntd.0011992 (PMC10927086; doi:10.1371/journal.pntd.0011992)
Supplement: Attachment — Submitted filename: Response to reviewers.pdf [file pntd.0011992.s004.pdf]

Response to the Reviewers

We would like to thank the Editor and the Reviewers for the time spent on reviewing our manuscript, their comments were valuable and helped us to improve the paper. Below is a point-by-point response to the reviewers' suggestions and questions.

Reviewer #1:

1. In light of the following sentence in the introduction section:" In recent years, both our group and other research teams have identified HDAC inhibitors that demonstrated effectiveness against S. mansoni and/or impacted the enzymatic activity of S. mansoni HDAC8 (SmHDAC8), (13–20)." the authors should include chemical structures of these 'identified HDAC inhibitors' in a new figure.

A new figure has been added with some examples of SmHDAC8 inhibitors reported in the literature (Fig.1)

2. How do the authors justify four significant figures for their reported LD50 values in Figure 1?

As pointed out by the reviewer, the uncertainties associated with the reported means did not deliver to that degree of accuracy; therefore, we modified the values in the Table embedded in New Figure 2

3. The following sentence: "However, we cannot exclude that the NF2839 and the other quinolone-based compounds are impacting parasite viability and/or eggs viability and maturation also through inhibition of other HDACs." unjustifiably assumes that all these compounds are inhibiting various aspects of schistosome biology solely through HDAC inhibition. These compounds, very likely, are acting by multiple mechanisms, only one of which may be HDAC inhibition. For example, was there any correlation between HDAC inhibition and schistosome inhibition for these compounds?

We modified the sentence including the possibility of off-target effects, even though our results on tubulin and histone acetylation points toward a possible engagement of HDACs

4. A consistent number of significant figures should be used for the data in Table 2.

We agree with the reviewer as some of the uncertainties associated with the sample means were not reliable; we amended the error; Table 2 has been revised and reports the uncertainties in a more consistent frame.

5. The authors should include cytotoxicity data for all the compounds reported in this study. Without these data, the reader has no idea if these compounds have any antischistosomal selectivity.

We agree with the reviewer that the cytotoxicity data are important. Viability assays have been performed on murine and human fibroblasts and a paragraph has been included in the revised version of the manuscript. The data are in a new Table S2

6. Did the authors generated any in vitro DMPK data for these compounds?

We did not perform in vitro DMPK studies; however in silico prediction of selected drug-like properties have been previously reported in Relitti et al, 2021. These data are in a new Table S1 and a paragraph has been included in the revised version of the manuscript.
