# Supplementary material for: Effects of structurally distinct human HDAC6 and HDAC6/HDAC8 inhibitors against S. mansoni larval and adult worm stages
Source: PLoS Negl Trop Dis. 2024 Feb 28;18(2):e0011992. doi: 10.1371/journal.pntd.0011992 (PMC10927086; doi:10.1371/journal.pntd.0011992)
Supplement: Attachment — Submitted filename: Response to reviewers.pdf [file pntd.0011992.s005.pdf]

Response to the Reviewers

We would like to thank the Editor and the Reviewers for the time spent on reviewing our revised version of the manuscript.

Supporting information Data S1 including the numerical values that were used to generate graphs (Fig. 2A, Fig. 2B) and histograms (Fig. 3) have been uploaded.

Table 2 with revisions in track change and Table 2 have been uploaded.

All Figures with PACE check have been uploaded with the manuscript, Tables and Supporting information

Reviewer #1: Despite their claims in the response to the initial manuscript review, the authors did not correct their inconsistent and faulty use of significant figures.

I am not sure to understand this Review comment.

To the previous objections:

1) How do the authors justify four significant figures for their reported LD50 values in Figure 1?

We modified the values in the Table embedded in the New Figure 2

2) A consistent number of significant figures should be used for the data in Table 2.

Table 2 was revised and reported the uncertainties in a more consistent frame for each independent enzymatic activity assay (hHDAC1, hHDAC6, hHDAC8, SmHDAC8), the values had a consistent number of significant figures in each column.

I guess that a common number of significant figures in the overall Table 2 is requested. In this revised version Table 2 indicates only the first significant figure after decimal point for all enzymatic assays, and when a zero is present the second is also shown.
